# Emissions-weighted carbon price: sources and methods

**DOI:** 10.1038/s41597-024-03121-6

**Published:** 2024-09-18

**Authors:** Geoffroy Dolphin, Magnus Merkle

**Affiliations:** 1 Resources for the Future, 1616 P St NW, Washington, DC 20036 USA; 2 International Monetary Fund, 700 19th St NW, Washington, DC 20431 USA; 3 Norwegian University of Life Sciences, Elizabeth Stephansens v. 15, 1433 Ås, Norway

**Keywords:** Energy economics, Climate-change mitigation, Environmental economics, Political economy of energy

## Abstract

This note describes the sources and methods used to calculate the emissions-weighted carbon price (ECP), the average price applied to CO_2_ emissions across all sources of emissions within a territorial jurisdiction by all carbon pricing mechanisms in force. It provides a transparent summary of the stringency of carbon pricing mechanisms in force within a given jurisdiction and allows for a straightforward comparison of that stringency across jurisdictions. It also describes the methodology behind two closely connected calculations: (i) sector-level carbon prices (by IPCC categories of emissions by sources and by categories of economic activity, respectively), (ii) industry- and country-level carbon costs.

## Background & Summary

Carbon pricing has become a central instrument of several national and subnational jurisdictions’ climate change mitigation strategies. As of 2022, 46 national and 31 subnational jurisdictions had a carbon pricing mechanism. However, a metric summarizing their stringency consistently across jurisdictions is lacking.

This note describes the data and methods used to calculate the emissions-weighted carbon price (ECP), a sector or economy-wide average price on CO_2_ emissions. A major benefit is that it provides a methodology to measure sector- or economy-level average prices consistently across jurisdictions. To the best of our knowledge, the ECP data constitute the first centralized and systematic assessment providing a consistent description of carbon prices that simultaneously includes price information disaggregated at the sector(-fuel) level, extends back to 1990 to include price information for the earliest carbon pricing policies, and accounts for as many sector(-fuel) exemptions as accurately possible. The methodology and data currently available allow to readily expand the calculation to new national or subnational jurisdictions, should some of their emissions become subject to a carbon pricing mechanism, as well as to new greenhouse gases.

It is calculated for 46 national and 31 subnational jurisdictions (13 Canadian provinces and territories, 11 US states, and 7 Chinese provinces) over 1990–2022. The average World CO_2_ price is also calculated. For national jurisdictions, the emissions-weighted price accounts for the prices arising from carbon pricing instruments introduced in their respective subnational jurisdictions. For instance, the emissions-weighted price for the United States includes the prices arising from state-level carbon pricing mechanisms.

This dataset is made available as an open-source resource with the hope that it can be useful to the academic community and society at large, as well as benefit from feedback and contributions from a wider community. We also hope that the calculation of an average, economy-wide, price on CO_2_ emissions will prove useful to inform cross-country comparisons of carbon prices.

The source code, written in Python 3, raw data files, formatted dataset files, and scripts are available at Zenodo. The digital object identifier associated with this repository is: 10.5281/zenodo.12181508. Files are available under a CC BY-NC 4.0 license (https://creativecommons.org/licenses/by-nc/4.0/). A version of this manuscript describing an earlier version of the dataset is available at https://www.rff.org/publications/working-papers/emissions-weighted-carbon-price-sources-and-methods/.

## Methods

To compute the ECP, the following information, for each jurisdiction and disaggregated at the sector(-fuel) level, is used: (1) emissions of each type of greenhouse gas (in CO_2_e); (2) effective coverage by a carbon pricing instrument; (3) the nominal price applied to emissions (in 2021 USD/tCO_2_e).

The calculation of the ECP involves four steps. First, data on jurisdictions’ emissions (disaggregated by IPCC categories of emissions by sources and fuel type) is collected and brought into a standardized inventory structure that follows IPCC 2006 guidelines (*Buendia et al*.^1^). Second, the emissions data is used to calculate the corresponding emissions share in (i) a jurisdiction’s and (ii) world total emissions of a given greenhouse gas. For emissions of subnational jurisdictions, the emissions share in the associated national jurisdiction is also calculated. Third, emissions data is linked with scope and price data contained in the World Carbon Pricing Database. Both datasets are disaggregated at the level of IPCC categories of emissions by sources, which allows for a one-to-one linking using IPCC category codes as the linking key. Finally, jurisdiction- and world-level coverage and emissions-weighted price figures are calculated as per the formulas presented below.

The programming script executing those steps is available as an iPython notebook on the Zenodo repository referenced above. The notebook is labelled ecp_v3.ipynb This script builds on dependencies, which are available at ~/_code/compilation/_dependencies/. A list of these dependencies and their description is available in the Supplementary Information (SI5). The programming scripts and the data taken from the World Carbon Pricing Database are publicly accessible.

The methodology can account for pricing of emissions of all greenhouse gases and, ultimately, provide an estimate of the average price of emissions of all greenhouse gases. The methodology description below reflects that. However, at this stage, the calculation has been implemented for CO_2_ emissions only due to (i) ongoing extension of the World Carbon Pricing Database to greenhouse gases other than CO_2_ and (ii) further methodological developments required by the less granular emissions inventory data for other greenhouse gases.

We collect greenhouse gas emissions data for all national and subnational jurisdictions in the dataset and combine it into a single, standardized inventory for each jurisdiction. Disaggregated data on emissions in each jurisdiction is obtained from various sources. Since these sources do not all follow the same structure or sectoral nomenclature, we construct correspondences that map the data of each one onto the harmonized structure in this dataset.

This correspondence implies both a harmonization of measurement units for emissions data (e.g, from kt to Mt CO2e) as well as a concordance of emissions source categories to IPCC categories (2006 Guidelines). These mappings are contained in the files ipcc2006_iea_category_codes.csv and ipcc_map_subnat.py. For subnational jurisdictions, inventories do not include a breakdown by fuel type within each IPCC source category 1A. As a result, we adjust the structure of the carbon pricing data to match that of the emissions inventory. Specifically, the price associated with the emissions of one fuel category (Natural gas) is selected. This has no impact on the accuracy of the calculation as the price of CO_2_ emissions in all subnational jurisdictions currently included in this dataset is the same across fuel categories. Should this change, a different approach would be implemented, such as calculating the arithmetic average of the price across all three fuel categories.

The calculation also accounts for coverage of emissions by multiple pricing mechanisms (i.e., overlapping coverage). To that end, we use the overlap_mechanisms_*.csv file maintained as part of the World Carbon Pricing Database. The * is a wildcard substituting for either CO2, CH4, N2O or F-GASES. These files contain a list of carbon pricing mechanisms that overlap with each other at the IPCC category level (see Supplementary Information SI4). This allows us to calculate the portion of emissions, within each category, that is covered by multiple mechanisms and subtract that portion of emissions from the sectoral total of covered emissions.

### Coverage: emissions

To calculate coverage, we combine data on the sectoral scope of carbon pricing mechanisms with inventory emissions data. The total coverage is calculated as the sum of sector (*k*) and, for IPCC categories 1A, sector(*k*)-fuel (*j*) level data. It is expressed as a share of a jurisdiction’s total emissions of greenhouse gas *g*.

Formally, the total emissions covered by all carbon pricing mechanisms in force in jurisdiction *i* in year *t* is given by

$$Coverag{e}_{i,t}^{g}=\sum _{k,j,m}{s}_{i,t,k,j,m}^{g}-{\omega }_{i,t,k,j}^{g}$$

where

${s}_{i,t,k,j,m}^{g}$ is the sector(-fuel) specific coverage by pricing mechanism *m*, expressed as a share of total emissions, and ${\omega }_{i,t,k,j}^{g}$ is the (share of) emissions covered by more than one carbon pricing mechanism (i.e., the overlap).

Note that ${s}_{i,t,k,j,m}^{g}\equiv {I}_{i,t,k,j,m}\times \frac{{e}_{i,t,k,j}^{g}\times c{f}_{i,t,k,m}^{g}.}{{e}_{total}^{g}}$ where ${I}_{i,t,k,j,m}$ is an indicator variable taking value 1 if fuel *j* in sector *k* of jurisdiction *i* in year *t* is subject to pricing mechanism *m*, ${e}_{i,t,k,j}^{g}$ is emissions from fuel *j* in sector *k* of jurisdiction *i* in year *t*, ${e}_{total}^{g}$ is total jurisdiction emissions of greenhouse gas *g* and $c{f}_{i,t,k,m}^{g}$ is a mechanism-specific coverage factor (see below).

We use emissions data to calculate the shares of sector (or sector-fuel)-level emissions in total jurisdiction (national or subnational) and world emissions of gas *g*. For subnational jurisdictions, we also calculate the share of emissions of each sector in total emissions of the relevant national jurisdiction, allowing us to calculate national coverage and average price figures arising from subnational pricing mechanisms.

The coverage factor accounts for scope exemptions of emissions within sectors, i.e., the fact that some pricing mechanisms cover less than 100 percent of emissions from a given IPCC emissions source category. Such exemptions include emissions/plants excluded due to (i) compliance thresholds or (ii) coverage by another pricing mechanism. Calculation or encoding of these coverage factors is described in the Supplementary Information (SI3).

### ECP

The ECP is the emissions-weighted average of sector(-fuel) level carbon prices. Its calculation entails (1) calculation of (mechanism-specific) aggregation weights (*w*) ; (2) multiplication of the aggregation weights by the price (*p*) applied to emissions by a given mechanism; (3) summation over all mechanisms; (4) aggregation at sector or jurisdiction level.

Formally, the calculation of the ECP of jurisdiction *i* in year *t* for greenhouse gas *g* can be expressed as

$$EC{P}_{i,t}^{g}=\sum _{k,j,m}{p}_{i,t,k,j,m}^{g}\times {w}_{i,t,k,j,m}^{g}$$

where

${p}_{i,t,k,j,m}^{g}$ is the price applicable to sector *k* by mechanism *m*;

${w}_{i,t,k,j,m}^{g}$ is the sector(-fuel) specific aggregation weight and is defined as ${w}_{i,t,k,j,m}^{g}\equiv {s}_{i,t,k,j,m}^{g}$.

${w}_{i,t,k,j,m}^{g}$ can be calculated using total [gas] or GHG emissions, yielding average [gas] or average CO_2_e prices. Given that the current version of the ECP does not account for prices on non-CO_2_ greenhouse gases, average CO_2_e prices may underestimate the actual average price on greenhouse gs emissions.

The ECP is calculated at the jurisdiction level (either subnational or national) and for a synthetic “World” jurisdiction. This is done by using the appropriate corresponding value of ${e}_{total}^{g}$ in the expression of ${s}_{i,t,k,j,m}^{g}$ above. For instance, when calculating the world average price of CO_2_ emissions, ${e}_{total}^{g}$ is the world total emissions of CO_2_. Furthermore, for jurisdictions in which a carbon price exists in one or more of its subnational entities, this price is added to the national average, using weights reflecting the share of these subnational emissions in national total emissions.

For IPCC Energy sectors (category 1A), a sector-level average of fuel-level prices is also computed. In that case, the weights are the shares of sector-fuel emissions in total sector CO_2_ emissions.

The formula above allows for two straightforward extensions. First, the same methodology can be used to calculate average carbon prices for *ad hoc* groups of countries; ${e}_{total}^{g}$ is then total emissions of that country group. Second, it can be used to calculate the average price of emissions for *world sectors*.

The ECP can be calculated using time-varying or fixed-year weights. For the former, coverage data is matched with emissions shares using jurisdiction, year, sector (and fuel type) as keys. This implies that emissions shares vary from year to year. For the latter, a reference year is selected (e.g., 2019) and the corresponding emissions data frame is matched with scope and price data using jurisdiction, sector (and fuel type) as linking keys. The same emissions shares apply across all years. The data in the present dataset is calculated using the former approach.

### Sector-level Carbon Prices by IPCC Emissions Source and Economic Activity Category

The world carbon pricing database (WCPD) (*Dolphin & Xiahou*^2^) provides carbon prices by IPCC categories of emissions, following the 2006 IPCC Guidelines for National Greenhouse Gas Inventories. Detailed reports explaining the IPCC accounting framework are available online (IPCC, 2023). In addition, for IPCC 1A subcategories (Fuel combustion), the prices are further disaggregated into three fuel categories, namely coal, natural gas and oil. The WCPD provides data at up to 5-digit IPCC emissions category code. The total number of categories for each year and country is 77. To obtain IPCC category-level prices, we aggregate fuel-level prices using the share of fuel-level emissions in total IPCC category emissions as weights.

We also calculate carbon prices by categories of economic activity. Indeed, while IPCC guidelines are the internationally accepted structure for emissions accounting, it does not exactly match the structure of standard classifications of economic activities such as the *International Standard Industrial Classification of All Economic Activities* (ISIC) or the *statistical classification of economic activities in the European Community* (NACE), according to which most (macro)economic aggregates are structured. It is therefore typically difficult to combine carbon pricing data with standard economic data such as value added, employment, productivity, etc.

Combining data requires a concordance table to allocate emissions, structured by IPCC emissions source categories, to economic activity categories. This is, in essence, the key technical step offered by air emissions accounts developed and made available by, e.g., Eurostat. However, publicly available versions of air emissions accounts typically do not provide, for each economic activity category, the breakdown of emissions by IPCC emissions source category. Hence, we turn to the Global Resource Input-Output Assessment model (GLORIA)^3,4^, which provides an allocation of IPCC emissions source categories to economic activity categories. The disaggregation follows the IPCC 2006 classification, and therefore enables us to use a precise mapping process for imputing emissions-weighted carbon prices at the GLORIA sector resolution.

The industrial classification used in GLORIA is based on ISIC rev.4, and provided at 120 sector resolution for each year and country. Greenhouse-gas emission data is provided for each sector in a disaggregated format, consistent with the EU Emissions Database for Global Atmospheric Research (EDGAR)^5^.

We match WCPD data to GLORIA sectors using a process-based algorithm. GLORIA CO_2_ emissions data are provided in 73 IPCC categories per country-activity, therefore the first step is an aggregation of the WCPD data from 77 to 73 categories. Where low level subcategories map onto higher level categories, we impose an emissions-weighted average carbon price. As a second step, we multiply for each activity and country the IPCC categorical emissions with the categorical carbon prices and divide by total emissions. The result is a country-activity specific emission-weighted carbon price. GLORIA only covers 160 jurisdictions and 4 rest of the world aggregates. Where several ECP jurisdictions map onto one GLORIA jurisdiction, we calculate the emissions-weighted average for the GLORIA jurisdiction.

Two versions of the IPCC emissions source category-level prices are calculated, constant 2021 USD (fixed exchange rate, 2021) and current USD (variable exchange rate).

### Carbon Cost

As the scope of carbon pricing mechanisms expanded and their stringency increased, interest for relating carbon pricing mechanisms with macroeconomic variables (e.g., GDP) has grown. Therefore, building on economic activity-level carbon prices described above, we also provide estimates of the carbon cost (in USD per USD value added) associated with carbon pricing mechanisms for categories of economic activity as well as at the aggregate level. This is currently implemented for year 2021 and will be expanded to 1990–2022.

To that end, we combine data on value added, also available from GLORIA, with emissions and carbon prices at the level of economic activities described above to calculate the carbon cost. In practice, this involves, for each economic activity category, the multiplication of emissions intensity (CO_2_ per unit of value added) by the economic activity category-specific (IPCC-weighted) carbon price.

Finally, we note that the above approach can easily be modified to calculate the share of a country’s GDP covered by carbon pricing. Straightforwardly, this is obtained by the ratio of the sum of every economic activity category whose carbon price is strictly positive and GDP.

### Sources

Information about the mechanisms’ sectoral scope and prices has been collected as part of a separate effort and is available through the World Carbon Pricing Database (*Dolphin & Xiahou*^2^). This information is collected at the IPCC emissions sourcecategory-fuel level. The sectoral disaggregation follows the guidelines of the International Panel on Climate Change (IPCC, 2006). The full dataset is available at https://github.com/gdolphin/WorldCarbonPricingDatabase, and the methodology used to compile the dataset is described in a companion Data Descriptor, available at https://www.nature.com/articles/s41597-022-01659-x.

The emissions price used to calculate the ECP is total price including any potential rebate. Prices in the World Carbon Pricing Database are expressed in current local currency units (LCUs). All prices in this dataset are expressed in 2021USD/tCO_2_e. The conversion from current LCUs uses the 2021 LCU/USD exchange rate and the jurisdiction-specific cumulative rates of inflation (based on the GDP deflator of each jurisdiction). Exchange rates are obtained from the Bank of International Settlements and GDP deflators are obtained from the World Bank Development Indicators (https://databank.worldbank.org/reports.aspx?source=World-Development-Indicators). For subnational jurisdictions, national-level GDP deflators are used.

To ensure (methodological) consistency across years and jurisdictions, we prioritize sources that provide data for the largest possible number of jurisdictions and the longest period. For national jurisdictions, the main sources are the International Energy Agency’s Grenhouse Gas Emissions from Energy^6^ (https://www.iea.org/data-and-statistics/data-tools/greenhouse-gas-emissions-from-energy-data-explorer) and the Emissions Database for Global Atmospheric Research (EDGAR) (https://edgar.jrc.ec.europa.eu/dataset_ghg60) provided by the Joint Research Centre of the European Commission. Both sources have an extensive sectoral coverage. However, the former provides a more granular disaggregation of 1A Energy emissions—specifically, (i) a higher sectoral granularity and (ii) a breakdown of emissions by fuel type—whereas the latter provides more detailed information for source categories 2. IEA data follows IPCC 2006 sectoral disaggregation but does not provide IPCC source category codes. Hence, we create a mapping between IEA sectors (flows) and IPCC emissions source categories. This mapping is available upon request. Data from the IEA is proprietary and hence not disclosed.

For subnational jurisdictions, emissions data is taken from various sources. For consistency with the carbon price data and the national jurisdictions emissions data, we prioritize sources that follow the IPCC sectoral disaggregation. United States state-level emissions are obtained from Rhodium Group Climate Deck^7^; Canada province-level emissions are obtained from Environment and Climate Change Canada^8^; China province-level CO_2_ emissions are taken from Carbon Emission Accounts and Datasets (CEADS)^9^.

In most cases, the last year for which GHG inventory data is available is 2020. Coverage and price calculations for years 2021-2022 use 2020 emissions data but year-specific scope and price data.

Note that EDGAR ‘China’ emissions refer to “People’s Republic of China” emissions only and is consistent with the geographical scope of IEA GHG emissions data for China.

Value added as well as emissions data by economic activity, used for the calculation of GDP coverage and Carbon Cost, is taken from the Global Resources Input Output Assessment (GLORIA)^4,5^. The GLORIA database is a time series of multi-regional input-output (MRIO) tables that were constructed using the Global MRIOLab infrastructure. It is a homogenous multi-regional supply-use table (MR-SUT) featuring identical sector labels for both the industry and commodity sectors.

## Data Records

A version of the dataset (as of June 20, 2024) is available on Zenodo (*Dolphin & Merkle*^10^).

### Scope

The dataset contains data on 189 national and 94 subnational (50 US states, 13 Canadian provinces and territories, 31 Chinese provinces, autonomous regions and municipalities) jurisdictions from 1990 to 2022.

All IPCC emissions source categories are included.

The ECP and other data provided currently exclusively account for prices on CO_2_ emissions. This reflects both historical policy developments (the first carbon pricing mechanisms applied to CO_2_ emissions only) and data constraints (cross-jurisdiction consistent emissions inventories for non-CO_2_ GHG are not always available at the required level of sectoral disaggregation).

This does not currently constitute a significant limitation, as most existing carbon pricing mechanisms apply exclusively to CO_2_ emissions. Only some mechanisms, such as the EU ETS and the Spanish tax on HFCs, do not. An extension of the dataset to non-CO_2_ gases is ongoing.

### Coverage

The database contains jurisdiction-level data on the coverage of CO_2_ emissions by carbon pricing mechanisms in the file tot_coverage_jurisdiction_CO2.csv. This file contains aggregate jurisdiction-level and World coverage of CO_2_ emissions by carbon pricing mechanisms for 1990–2022. This file presents, for each jurisdiction, the share of emissions covered by (i) carbon taxes, (ii) emissions trading systems (ETSs), or (iii) the combination of both. For each of these instruments, the coverage figures are calculated as a share of (i) total GHG and total CO_2_ emissions and (ii) total jurisdiction or total world emissions. Furthermore, for subnational jurisdictions, emissions as a share of the relevant national jurisdiction are also calculated. This yields 18 variables. A list of variables and their description is included in the Supplementary Information (SI1).

### ECP

The file ecp_CO2.csv contains the jurisdiction-level average carbon price. Following from the calculation of coverage shares, the ECP is calculated separately for carbon taxes and ETSs and the combination of both. It is also calculated using GHG and CO_2_ shares.

### Sector-level prices

Files containing sector-level prices are available at the following locations of the repository:

- By IPCC emissions source category:
  - /_dataset/ecp/ipcc/ecp_sectors/constantPrices/FixedXRate/ecp_sector_CO2.csv
  - /_dataset/ecp/ipcc/ecp_sectors/currentPrices/FlexXRate/ecp_sector_CO2.csv
- By economic activity category:
  - /_dataset/ecp/industry/ecp_gloria_sectors/edgar_based/constantPrice/FixedXRate/ecp_gloria_industry_CO2.csv
  - /_dataset/ecp/industry/ecp_gloria_sectors/edgar_based/currentPrice/FlexXRate/ecp_gloria_industry_CO2.csv

## Technical Validation

The calculation involves several steps, including quality assurance.

### Data quality

All sources upon which the dataset relies are either publicly accessible and have undergone a peer-review process (e.g., data carbon pricing) or provided by third-party organization following robust quality assurance processes (e.g., emissions data).

### Quality assurance

The entire dataset generation pipeline is hosted on GitHub. This includes both the raw data and the final dataset files, as well as the Python files implementing the transformation of the former into the latter. All modifications to the raw data files are executed on separate development branches of the repository and reviewed before integration into the main branch. The consistency of the change or update with the original data source is checked upon review.

## Usage Notes

### Planned update and future extensions

The dataset is under continuous development. Suggestions for and contributions to the extension of the dataset to other features of carbon pricing mechanisms are welcome. The next update of the dataset will focus on the following:

1. Update of data to the latest year to reflect institutional design and price changes pertaining to mechanisms and information on mechanisms established since the last release.
2. Adjustment of the methodology and emissions data to account for prices on non-CO_2_ greenhouse gases.
3. Calculation of economic sector-level carbon prices using OECD emissions inventories. The current ones are based on EDGAR inventories.
4. Addition of economic sector-level price series accounting for free allowances in the EU ETS.
5. Calculation of coverage and ECP figures for two Japanese municipalities: Saitama and Tokyo.

The dataset is available under a CC-BY-NC licence with non-commercial restrictions required due to the use of input data from a third party (International Energy Agency, IEA) and their requirements for derived data.

## Supplementary information

Supplementary Information

## Data Availability

All code is written in Python 3 or R programming languages. All files, including code necessary to the compilation of the dataset are available on the following GitHub repository: https://github.com/g-dolphin/ECP.
