# Supplementary material for: Emissions-weighted carbon price: sources and methods
Source: Sci Data. 2024 Sep 18;11:1017. doi: 10.1038/s41597-024-03121-6 (PMC11411118; doi:10.1038/s41597-024-03121-6)
Supplement: Supplementary file 1 — Supplementary Information [file 41597_2024_3121_MOESM1_ESM.docx]

Supplementary Information

Table of Contents

[SI1 Dataset content 2](#_Toc116055772)

[SI2 Dataflow representation 3](#_Toc116055773)

[SI3 Coverage factors 3](#_Toc116055774)

[SI4 Mechanisms overlap 3](#_Toc116055775)

[SI5 Dependencies 4](#_Toc116055776)

# SI1 Dataset content

Table 1 Description of variables in file tot_coverage_CO2.csv

| Variable name | Description |
| --- | --- |
| cov_tax_CO2_jurCO2 | CO_2_ emissions covered by a carbon tax as a share of jurisdiction total CO_2_ emissions. |
| cov_tax_CO2_wldCO2 | CO_2_ emissions covered by a carbon tax as a share of world total CO_2_ emissions. |
| cov_tax_CO2_supraCO2 | CO_2_ emissions covered by a carbon tax as a share of national jurisdiction total CO_2_ emissions. |
| cov_ets_CO2_jurCO2 | CO_2_ emissions covered by an ETS as a share of jurisdiction total CO_2_ emissions. |
| cov_ets_CO2_wldCO2 | CO_2_ emissions covered by an ETS as a share of world total CO_2_ emissions. |
| cov_ets_CO2_supraCO2 | CO_2_ emissions covered by an ETS as a share of national jurisdiction total CO_2_ emissions. |
| cov_all_CO2_jurCO2 | CO_2_ emissions covered by either a carbon tax or an ETS as a share of jurisdiction total CO_2_ emissions. |
| cov_all_CO2_wldCO2 | CO_2_ emissions covered by either carbon taxes or an ETS as a share of world total CO_2_ emissions. |
| cov_all_CO2_supraCO2 | CO_2_ emissions covered by either carbon taxes or an ETS as a share of national jurisdiction CO_2_ emissions. |

Note: total emissions used to calculate emissions shares exclude those from Land Use and Land Use Change and Forestry.

Table 2 Description of variables in file ecp_CO2.csv

| Variable name | Description |
| --- | --- |
| ecp_ets_jurCO2_usd_k | Emissions-weighted average price on emissions covered by an emissions trading system (ETS).  *Weights*: share of jurisdiction total CO_2_ emissions.  *Unit:* 2021 constant (‘k’) US Dollars (‘usd’) |
| ecp_tax_jurCO2_usd_k | Emissions-weighted average price on emissions covered by a carbon tax.  *Weights*: share of jurisdiction total CO_2_ emissions.  *Unit:* 2021 constant (‘k) US Dollars (‘usd’) |
| ecp_all_jurCO2_usd_k | Emissions-weighted average price on emissions covered by either a carbon tax or an ETS.  *Weights*: share of jurisdiction total CO_2_ emissions.  *Unit:* 2021 constant (‘k’) US Dollars (‘usd’) |
| ecp_ets_supraCO2_usd_k  (Subnational jurisdictions only) | Emissions-weighted average price on emissions covered by an emissions trading system (ETS).  *Weights*: share of national jurisdiction total CO_2_ emissions.  *Unit:* 2021 constant (‘k’) US Dollars (‘usd’) |
| ecp_tax_supraCO2_usd_k  (Subnational jurisdictions only) | Emissions-weighted average price on emissions covered by a carbon tax.  *Weights*: share of national jurisdiction total CO_2_ emissions.  *Unit:* 2021 constant (‘k) US Dollars (‘usd’) |
| ecp_all_supraCO2_usd_k  (Subnational jurisdictions only) | Emissions-weighted average price on emissions covered by either a carbon tax or an ETS.  *Weights*: share of national jurisdiction total CO_2_ emissions.  *Unit:* 2021 constant (‘k’) US Dollars (‘usd’) |

*Table 3 Description of variables in files ecp_gloria_finaldem_CO2.csv and ecp_gloria_industry_CO2.csv*

| Variable name | Description |
| --- | --- |
| ecp | (Emissions-weighted) price of CO_2_ emissions applicable to the economic activity category (i.e., industry)  *Unit:* 2021 constant US Dollars |
| CO2 | CO_2_ emissions (kt) of the economic activity category (i.e., industry). |

Note: total emissions used to calculate emissions shares exclude those from Land Use and Land Use Change and Forestry.

# SI2 Dataflow representation


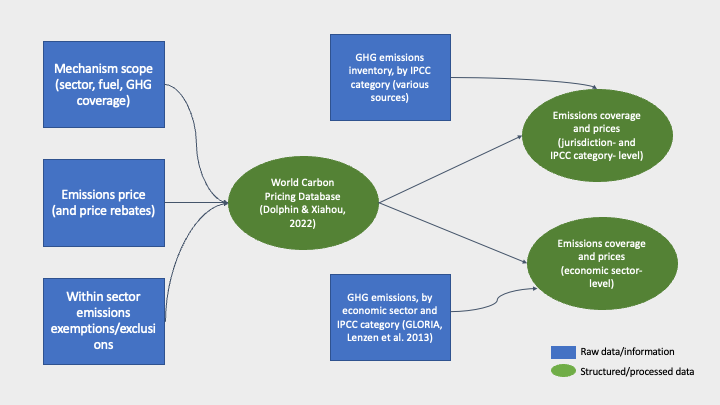


# SI3 Coverage factors

Our assessment of emissions covered by carbon pricing mechanisms within each jurisdiction is based on inventory data, which, in theory, includes *all* emissions of a given sector. However, some emissions of specific sectors may be excluded from the scope of a given carbon pricing mechanism. To account for such exclusions, we calculate or encode sector-specific coverage factors.

This coverage factor is an initial attempt to account for all exemptions that allow firms in specified sectors or meeting certain conditions within those sectors to exempt part of their emissions from the tax or waive their obligation to surrender allowances for these emissions. This includes, for instance, compliance thresholds above which firms (or plants) become liable for the carbon tax or must surrender emissions allowances or outright waivers on some proportion of emissions within a sector. The latter occurs when the emissions might be covered by two mechanisms and the liability is waived for one of the two.

See section 4.1.4 of *World Carbon Pricing Database: Sources and Methods* for further details.

# SI4 Mechanisms overlap

Within a jurisdiction, two mechanisms typically do not apply to the same sectors; that is, they have no sectoral overlap in coverage. Mechanisms do sometimes overlap at the sectoral level, but this overlap does not extend to actual emissions within those sectors, as the mechanisms are designed to apply to different emissions within them. For instance, for countries participating in the EU ETS, their national carbon tax is designed to cover only emissions from installations that are not participating in the EU ETS.

However, an overlap sometimes exists. Overlap between carbon pricing mechanisms is accounted for by maintaining a csv file recording the bilateral overlap between mechanisms, at the sector level.

# SI5 Dependencies

Directory: ~/_dependencies/dep_ecp

| **Label** | **Description** |
| --- | --- |
| pkgs_and_directories.py | Defines all paths and loading all dependencies necessary to the execution of the script ecp_v3.ipynb. |
| bis_xRate.py | Reads and processes exchange rate data (LCU/USD) from the Bank of International Settlement. |
| jur_names_concordances.py | Contains all concordances between the names of jurisdictions in the source datasets and the jurisdiction nomenclature in this dataset. |
| ipcc_map_subnat.py | Contains a mapping between categories of subnational emission inventories (Canada, China, United States) and IPCC2006 categories. |
| ecp_v3_gen_func.py | Contains small functions used in the main script. |
| ecp_v3_overlap.py | Contains a function loading and processing the data on overlapping carbon pricing mechanisms for use in the calculations. |
| inventory_preproc_nat.py | Contains the functions that generate the harmonized emission inventory for national jurisdictions. |
| inventory_preproc_subnat.py | Contains the functions that generate the harmonized emission inventory for subnational jurisdictions. |
| ecp_v3_inventory_share_func.py | Calculates the share of category-specific emissions in total emissions. |
| ecp_v3_coverageFactors.py | Loads and processes the coverage factors data for use in the calculations. |
| ecp_v3_coverage.py | Calculates coverage of emissions by carbon pricing mechanisms. |
| ecp_v3_curr_conv.py | Converts emission prices from local currency units to 2021 USD. |
| ecp_v3_weightedAverage.py | Calculates emissions-weighted average price of emissions. |

Directory: ~/_dependencies/dep_ccost

| **Label** | **Description** |
| --- | --- |
| gloriaProcessing.py | Processes output and value added data from the Global Resource Input-Output Assessment dataset. |
| pricingProcessing.py | Reads and processes economic activity-level carbon prices. |
